# Impact of system-level changes and training on alcohol screening and brief intervention in a family medicine residency clinic: a pilot study

**DOI:** 10.1186/1747-597X-8-9

**Published:** 2013-02-28

**Authors:** James Aaron Johnson, James Paul Seale, Sylvia Shellenberger, Maribeth Hamrick, Robert Lott

**Affiliations:** 1 Department of Family Medicine, Mercer University School of Medicine and Medical Center of Central Georgia, 3780 Eisenhower Parkway, Suite 3, 31206, Macon, GA, Georgia; 2 Department of Medicine 1161, Vanderbilt University Medical Center, 21st Avenue S, 37232, Nashville, TN, USA; 3 Department of Dermatology, New York Medical College, 1901 1st Ave, 10029, New York, NY, USA

**Keywords:** Alcohol screening, Medical education, Resident training, Brief intervention, Chart review

## Abstract

**Background:**

Although screening and brief intervention (SBI) are effective in reducing unhealthy alcohol use, major challenges exist in implementing clinician-delivered SBI in primary care settings. This 2006–2007 pilot study describes the impact of systems changes and booster trainings designed to increase SBI rates in a family medicine residency clinic which annually screened adults with a self-administered AUDIT-C questionnaire and used paper prompts to encourage physician interventions for patients with positive screens.

**Methods:**

Investigators added the Single Alcohol Screening Question (SASQ) to nursing vital signs forms, added a checkbox for documenting brief interventions to the clinicians’ outpatient encounter form, and conducted one-hour nurse and clinician booster trainings. Impact was measured using chart reviews conducted before implementing systems changes, then six weeks and six months post-implementation.

**Results:**

At all three time points screening rates using AUDIT-C plus SASQ exceeded 90%, however AUDIT-C screening decreased to 85% after 6 months (p=.025). Identification of unhealthy alcohol users increased from 4% to 22.9% at six weeks and 18.8% at six months (p=.002) using both screens. Nursing vital signs screening using the SASQ reached 71.4% six weeks after implementation but decreased to 45.5% at six months. Changes in clinician brief intervention rates did not achieve statistical significance.

**Conclusions:**

This is the second study reporting sustained primary care alcohol screening rates of more than 90%. Screening patients with SASQ and/or AUDIT-C identified a higher percentage of patients with unhealthy alcohol use. Dissemination of effective strategies for identifying unhealthy alcohol users should continue, while future research should focus on identifying more effective strategies for increasing intervention rates.

## Background

There is strong evidence for the efficacy of screening and brief interventions (SBI) in reducing unhealthy alcohol use, particularly in primary care settings [1,2]. A recent study comparing preventive services found SBI to be the third highest in terms of preventable burden of disease and cost-effectiveness, with high potential for reducing both medical and societal costs related to unhealthy alcohol use [3] as well as alcohol-related health risks [4]. As a result of SBI’s efficacy, the US Preventive Services Task Force recommends screening all primary care patients and providing behavioral counseling interventions (typically including feedback on their drinking, advice to reduce consumption, and if possible, negotiating a lower drinking goal) to reduce unhealthy alcohol use [5].

Because current levels of SBI delivery are among the lowest among comparable preventive services, the Substance Abuse and Mental Health Services Administration has mounted a major initiative supporting residency training in SBI and referral to treatment (SBIRT) for both alcohol and drugs. [6] While several previous training efforts have resulted in modest increases in rates of advising patients to reduce drinking among practicing physicians [7-9] and in residency training programs [10-13], several recent studies have found major challenges to increasing primary care clinicians’ brief intervention rates despite investment of significant time and resources [14,15].

The Healthy Habits Project (HH1) was a pilot program which resulted in significant increases in alcohol SBI in a family medicine residency training clinic during 2002–2003 [10]. Details of implementation methods have been previously published [11]. Briefly, clinicians participated in a three-hour training which included a didactic component and skills-based training. The project’s approach to SBI service delivery, modeled after the WHO’s PHEPA Project and the University of Connecticut’s Cutting Back Project [7,8,16], included use of a modified AUDIT-C questionnaire for alcohol screening, inclusion of the AUDIT-C on a self-administered paper health habits questionnaire distributed by clinic receptionists, the scoring of the initial alcohol screen by nurses who also gave screen-positive patients paper AUDIT questionnaires to complete for further assessment, the placement of intervention brochures and other SBI materials at physician workstations throughout the clinic, and providing residents and nurses with performance feedback regarding brief intervention (BI) rates on a monthly basis. Low initial screening rates were addressed by incorporating the AUDIT-C questions into annual self-administered patient information updates required by guidelines of the Joint Commission on Accreditation of Hospital Organizations (JCAHO), distributed by receptionists and completed in the waiting room. After incorporation of AUDIT-C questions into this update form, overall screening rates reached 82%. Alcohol screening was included among other survey questions required by JCAHO because collection of required JCAHO information is a high priority for the clinic’s sponsoring hospital, and staff regularly receive feedback designed to ensure that JCAHO requirements are met.

After conclusion of the study, investigators identified several areas for potential project improvement: rates of unhealthy alcohol use (8%) were well below the 28% reported by the National Epidemiologic Survey on Alcohol-Related Conditions [17], percentages of unhealthy drinkers receiving brief interventions were less than 50%, and chart reviews revealed limited documentation of SBI services provided [11]. This pilot study examines the impact of quality improvement efforts involving both systems changes and booster trainings to address barriers to SBI service delivery. It was hypothesized that the interventions would increase the rates of patients screened, unhealthy drinkers identified and BIs documented.

## Methods

Healthy Habits II (HH2) was designed and implemented in June 2006 using three specific quality improvement efforts described below:

1) Screening protocol: AUDIT-C annual questionnaire screening as conducted in HH1 continued. In an attempt to increase alcohol screening rates and identification of unhealthy drinkers, clinic procedures were modified to integrate verbal administration of single screening questions for tobacco use and unhealthy drinking into nursing vital signs. For alcohol screening, nursing vital signs templates were modified to include the SASQ, a validated measure for identifying unhealthy drinking advocated by the NIAAA Clinician’s Guide [17,18], as well as a simple method for recording responses. Nurses were requested to ask the SASQ at every visit as part of patient vital signs, and to ask all screen-positive patients to self-administer the paper AUDIT questionnaire and give it to their physician. Training sessions emphasized to nurses that the SASQ had been carefully worded and validated and should be asked exactly as written. Sessions included skills practice and discussions of how to manage problems encountered during screening.

2) Modification of clinical encounter charting forms: In an attempt to increase documentation of physician BIs, physicians’ clinical encounter forms in the clinic’s paper chart system were modified to include checkboxes for documenting brief interventions for alcohol and tobacco. These checkboxes were included in the section of the form where physicians documented their treatment plans. All clinicians were accustomed to routine review of their charts for quality control purposes, but were not specifically informed that this section of the chart would be reviewed.

3) Booster training for nurses and residents: In an attempt to improve rates of brief intervention, residents received a one-hour SBI booster training session timed to coincide with implementation of single question screening. All residents in the residency program had previously received a three-hour skills-based seminar, similar to the HH1 training, as part of their orientation at the beginning of their residency training. The HH2 conference reinforced the importance of BI for unhealthy drinking, taught residents how to interpret responses to the SASQ, reviewed procedures for scoring AUDIT forms and conducting brochure-based BIs, and encouraged residents to use the new checkboxes stating “Advised to stop smoking” and/or “Advised to quit drinking/cut back” when brief interventions were performed. Faculty received an abbreviated orientation to changes in the charting form during faculty meetings.

**Figure 1:**
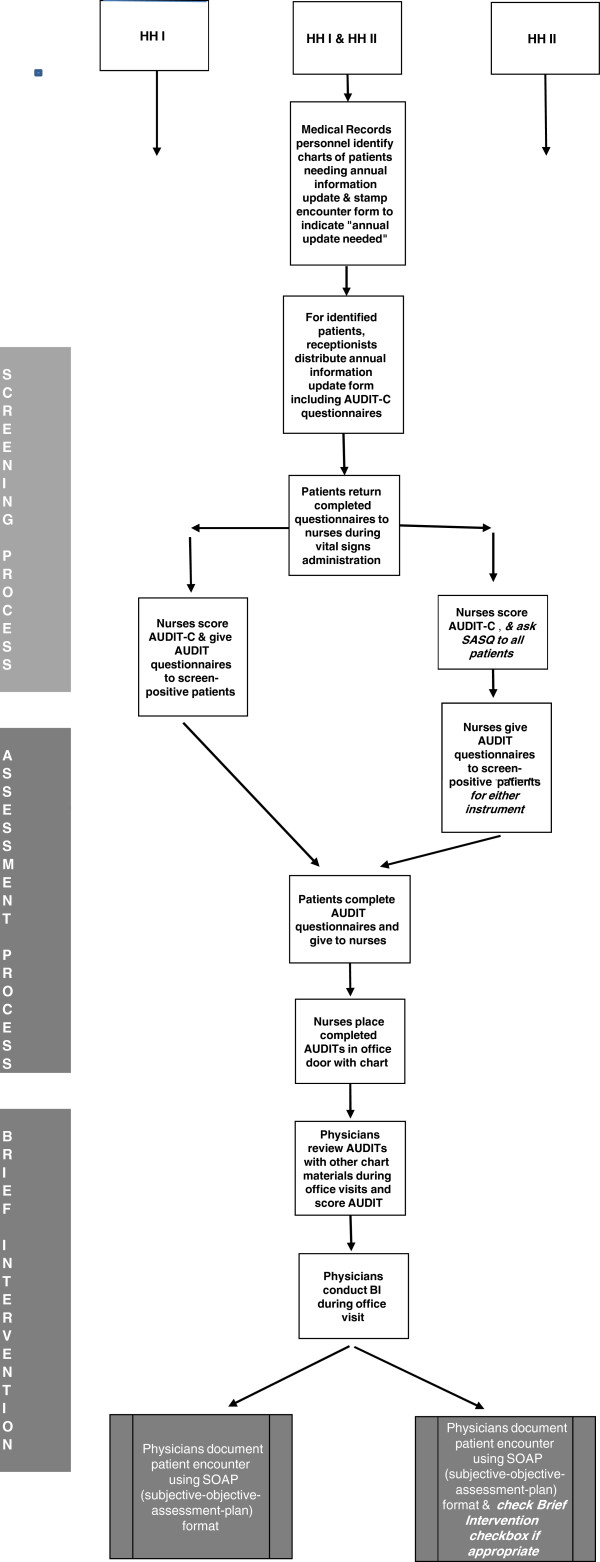
Intervention steps for HHI and HHII.

Figure 1 provides a side by side comparison of the procedural differences for conducting SBI in HHI and HHII.

### Instruments

The AUDIT-C is a three-question validated instrument for detecting at-risk and problem drinking [19-21], taken from the ten-question Alcohol Use Disorders Identification Test, developed by the World Health Organization (WHO) [22]. Questions 1 and 2 assess the quantity and frequency of alcohol consumption. Question 3 assesses the frequency of high-volume drinking days. For this study, Question 3 was modified to ask “How often do you have 4 or more drinks on one occasion?” rather than “How often do you have 6 or more drinks on one occasion?” This modification, which was also used in the “Cutting Back” study [7,8], was made based on recommendations in WHO guidelines for use of the AUDIT [23], due to differences in the larger size of the U.S. standard drink (14 g) as compared to the 10 g standard drink used in the original AUDIT validation. Using a cutoff score of 4 or higher, U.S. studies have found the AUDIT-C to have sensitivity ranging from 76% to 86% and specificity ranging from 72% to 80% in detecting unhealthy alcohol use [19,24].

The NIAAA SASQ is a single question validated instrument for detecting at-risk and problem drinking advocated for universal screening in primary care by the U.S. National Institute for Alcohol Abuse and Alcoholism [“How many times in the past year have you had…5 or more drinks in a day (for men) OR 4 or more drinks in a day (for women)”] [17]. Using a cutoff point of one or more times in the past year as a positive screen, SASQ has a sensitivity of 82% and specificity of 79% in detecting unhealthy alcohol use in primary care patients. [18]

### Measurement methods

This study was approved by the Mercer University and Medical Center of Central Georgia Institutional Review Boards. Due to limited resources and the pilot nature of this study, a series of three reviews of approximately 100 patient charts was conducted on consecutive adult patients seen by residents in the residency clinic at specific time points. A pre-intervention chart review (n=95) was conducted, beginning one month prior to implementation of HH2. Post-intervention chart reviews were conducted beginning six weeks (n=93) and six months (n=100) after implementation. These intervals were chosen based on previous observation that quality improvement interventions often produce an immediate change in staff behavior that dissipates over time. Chart reviews assessed whether the 1) AUDIT-C and/or single alcohol screening question were completed, 2) the patient’s score on these instruments was positive or negative, and 3) there was evidence in either the clinician’s progress notes or checkboxes that a brief intervention was conducted by the physician. BI was defined as any comment indicating alcohol education was given, patients were given advice to reduce or stop drinking, or evidence of referral for detoxification or treatment. Previous studies indicate that clinicians frequently neglect to document both alcohol diagnoses and brief interventions [10,25].

### Analyses

This study design used chi-square tests to identify significant changes across the study’s three time points in the percentage of patients being screened by the AUDIT-C and/or SASQ, the percentage screening positive for unhealthy alcohol use, and for those screening positive, the percentage receiving a physician-delivered brief intervention.

## Results

In each of the three chart review samples, the average patient was over 40 (41.4 years, 48.1 years, and 48.4 years, respectively), a majority of patients were African American and females comprised approximately two thirds of those sampled (72.6%, 59.1%, 58.0%, respectively). Gender and ethnicity composition of study patients was similar to published data regarding the clinic’s overall demographic pattern [26]. A comparison of demographic characteristics across the three time points (see Table 1) shows a significantly lower average age among charts sampled before implementation than 6 weeks and 6 months post-implementation. Gender and race/ethnicity were not significantly different across the three time points.

**Table 1:** Participating patient demographics across each enrollment period

	**Baseline (n=95)**	**6-weeks (n=93)**	**6- months (n=100)**	**Chi-square value (p-value)**
% Female	72.6%	59.1%	58.0%	5.455 (.065)
Race/Ethnicity				5.863 (.210)
- African American	53.6%	50.0%	64.0%	
- Caucasian	44.3%	48.9%	33.0%	
- Other	2.1%	1.1%	3.0%	
Mean Age in Years (SD)^a^	41.4 (15.8)	48.1 (13.7)	48.4 (13.4)	7.268(.001)

^a^ – One-way ANOVA performed for differences in means yields F-statistic = 7.268 and p=.001.

Table 2 compares rates of screening, detection of unhealthy alcohol use, and brief interventions for the three time points. Available data from the original Healthy Habits 1 study are included for comparison purposes. During the implementation period, the annual screening of patients using the AUDIT-C forms distributed by receptionists continued. The percentage of patients receiving the AUDIT-C at any of the data collection periods remained high, though a chi-square test indicates a statistically significant 10% decline between 6 weeks and 6 months post system change (p=.025). SASQ screening rates at six weeks post implementation were 71.4%, however this rate decreased to 45.4% after six months (p<.001). More than 90% of patients were being screened for unhealthy alcohol use using AUDIT-C and/or SASQ at all three time points, and at 6 months, the percentage of patients being screened by at least one of these instruments was significantly higher when compared to AUDIT-C alone (90.6% vs. 85.6%, df=95, p=.025).

**Table 2:** Screening, unhealthy drinking, and intervention rates across each enrollment period

		**HH1 (2003)**	**Baseline (n=94)**	**6-weeks (n=86)**	**6- months (n=97)**	**Chi-square Value**^**a **^**(p-value)**
Screening Rate	AUDIT-C	n/a	94.7%	95.3%	85.6%	7.393 (p=.025)
	SASQ	---	--	71.4%	45.4%	13.123 (p<.001)
	AUDIT-C and/or SASQ	n/a	94.7%	97.6%	90.6%	3.991 (p=.136)
Patients screened		**n=3014**	**(n=91)**	**(n=84)**	**(n=92)**	
Positive Rate	AUDIT-C	8.0%	4.4%	14.3%	14.1%	5.962 (p=.051)
(% of Screened)	SASQ	-------	--	22.4%	13.6%	1.326 (p=.250)
	AUDIT-C and/or SASQ	8.0%	4.4%	22.9%	18.8%	13.004 (p=.002)
Screen positives		**n=241**	**(n=4)**	**(n=21)**	**(n=18)**	
Intervention Rate (% of Screen Positives)		47.7%	25.0%	52.4%	61.1%	1.736 (p=.420)

^a^ Chi-square test statistic with 2 ° of freedom.

Among patients screened (n=267), screen positive rates were comparable between AUDIT-C and SASQ at both post-implementation time points. The total percentage of patients identified as screen positive by SASQ and/or AUDIT-C was significantly higher than the percentage identified by the AUDIT-C alone at 6 weeks (22.9% vs. 14.3%, df=82, p=.004). At 6 months, the difference in screen positive rates was not statistically significant (18.8% vs. 14.1%, df-68, p=.083). Using the combination of AUDIT-C and/or SASQ to screen patients resulted in a significant increase in the identification of unhealthy drinking in the post-implementation periods to 22.9% and 18.8%, respectively (p=.002), when compared to the baseline screen-positive rate (4.4%). Though the data in Table 2 appear to show an increase in brief intervention rates from baseline (25%) to 6 weeks (52.4%) and 6 months (61.1%), the change between baseline and post implementation measures is not statistically significant (df=68, p=.083), perhaps due to limited sample size and the small number of positive screens at pre-implementation (n=4).

## Discussion

### Screening rates

HH2 interventions did not significantly increase the percentage of patients receiving alcohol screening, perhaps because baseline screening rates were already high, leaving little room for increase. While universal screening (i.e. 100%) is desirable, reaching 90+ percent of patients with one or more standardized screening instruments is an acceptable outcome and one that has not been replicated in many studies of SBI implementation.

### Detection of unhealthy alcohol use

Combined use of AUDIT-C and/or SASQ detected higher numbers of unhealthy drinkers than either instrument alone. This finding is consistent with two recent validation studies which found higher sensitivity for detecting unhealthy alcohol use by combining use of a single question to screen for heavy drinking with AUDIT screening [27,28]. The percentage of unhealthy drinkers identified by the SASQ varied over time. During the project’s initial 6 weeks, the SASQ achieved the highest screen-positive rate measured by any single instruments alone--22.9%, a rate which is only slightly lower than the 28% rate found in NESARC survey [17]. For reasons that are unclear these rates were not sustained. Possible explanations include nurses changing the way questions were asked in light of patient dissatisfaction or patients changing their answers to avoid having to undergo further assessment and BI. Another curious finding from this study is the low screen-positive rate on the AUDIT-C (4.4%) during the baseline period. Demographic differences between the baseline chart review and subsequent chart reviews, including the lower mean age of patients and higher percentage of female participants, could account in part for this difference. The analyses presented here did not allow for control of demographics as potentially confounding variables. Previous research, conducted in primary care settings, across diverse groups of patients has found single alcohol screening questions and the AUDIT-C to have similar sensitivity when both were administered verbally in confidential studies [18,21,24,27,29]. Bradley et al. previously compared verbal administration of the AUDIT-C in the clinical setting with self-administered AUDIT-C questionnaires collected through a mail survey, and found clinical screening to be less effective in identifying unhealthy drinkers [30]. This study is one of the first to compare verbal administration of a single question screen with written administration of the AUDIT-C, and found similar levels of detection of unhealthy drinking. While reasons for this finding are unclear, it is possible that it is easier to perform single question screening with high levels of fidelity in the clinical setting, or that patients are less defensive in honestly answering the single question screen. Incorporating both AUDIT-C and SASQ instruments into the clinic increased the detection of unhealthy drinkers by 4.7-8.6% when compared with AUDIT-C alone and by 0.5-5.2% when compared to SASQ alone. Larger longitudinal studies are needed to determine whether these differences are sustained over time and whether the increased number of patients detected by using two instruments with overlapping domains and similar sensitivity and specificity warrants the additional cost incurred. Simply adding the SASQ to the AUDIT-C in written form, which would eliminate the use of additional time during nursing vital signs, is a potential screening option to be tested in future studies.

### Impact on BI rates

During this pilot project, BI rates were more than twice those at baseline, returning to levels slightly above those reported in HH1. While these increases in BI rates did not achieve statistical significance, a retrospective power analysis indicates that the study was underpowered to detect this level of difference in BI rates. With particularly low rates of screen positives at baseline, the sample of charts would need to be more than three times as large to detect a significant change in BI rates. With limited resources, a study of that size was not feasible.

This study utilized booster trainings and performance feedback to improve BI rates, in contrast to HH1, which had included an initial training plus performance feedback but no booster trainings. As the search continues for effective strategies for achieving and maintaining high BI rates, a potential area for future study is comparing the impact of booster trainings on BI rates versus performance feedback versus a combination of these two approaches.

### Systems issues

In an attempt to create a sustainable system that would consistently identify a high percentage of unhealthy drinkers, HH1 and HH2 tested attempts to link alcohol screening to two different “universal” procedures--annual self-administered information updates, which are required by JCAHO, and nursing vital signs, which are routinely performed on all patients. In this setting, use of annual self-administered screening questionnaires was the single approach which achieved the highest screening rate, with high levels of acceptability among both patients and clinic staff. Single question screening during nursing vital signs at every clinic visit proved to be problematic. While asking nurses to screen patients at every visit avoided the challenge of creating a reminder system that would prompt nurses at a specific interval (for example, once a year), both nurses and patients described dissatisfaction with verbal screening at every visit. While initial screening rates were high, over six months there were declines in both completion rates by nurses (71% to 45%) and in screen-positive rates (22% to 14%).

Reasons for the decline in self-administered questionnaire screening rates (from 93% to 85%) at 6 months are unclear, but could be related to the increased attention being given to verbal administration of the SASQ. Nonetheless, screen-positive rates were relatively high (14%) at both post-intervention measurement points and screening rates exceeded 85% at every time point, demonstrating both the effectiveness of linking alcohol screening to information required by JCAHO and high acceptance of this self-administered screening approach to both patients and clinic staff. This is the second study to achieve ongoing alcohol screening rates of 85% or higher. The Veterans Administration outpatient system achieved an annual AUDIT-C alcohol screening rate of 93% after adoption of a mandatory performance measure for alcohol screening in 2003 that also included training of quality managers, use of a computerized clinical reminder with automatic scoring of the AUDIT-C questionnaire, monitoring by both medical record reviews and patient satisfaction questionnaire, and financial incentives for high performance [31]. This study, conducted in a clinic without an electronic medical record, achieved these results by combining nurse and clinician training with a different kind of systems change—tying alcohol screening to a carefully-monitored systems process which was linked to an accreditation process that generates high levels of monitoring and compliance by many U.S. healthcare facilities. It is noteworthy that, in contrast to declines in BI rates, alcohol screening rates, which reached 82% by the end of HH1 [11], actually increased during the three years following the HH1 study, despite the absence of booster trainings and performance feedback.

### Limitations

Some differences observed in this study could be due to differences in patient demographics, rather than the project’s systems interventions. As a small pilot study in a single clinic, the results of the study may not be generalizeable to other primary care clinics. Larger multi-site studies are needed to determine if results are replicable. Likewise, clinic staff members were aware that their screening and brief intervention activity was being observed and documented. This could have resulted in a Hawthorne effect whereby the positive changes in screening rates were largely the result of study itself.

## Conclusions

This study indicates that alcohol screening using the SASQ and/or AUDIT-C can increase identification of unhealthy drinkers. Results of this study and others [30-32] suggest that effective strategies have now been developed that can achieve alcohol screening rates of 90% or higher, and that efforts to disseminate such strategies more widely are needed. Greater attention now needs to be directed to methods for increasing BI rates, which may require different strategies. BI rates in HH1 and HH2 compare favorably with most SBI efforts analyzed in a recent review by Williams et al. [32]. The drop in BI rates between HH1 and HH2, followed by SBI increases during this pilot project, suggest the value of interventions such as performance feedback and booster trainings in the face of the constant competing demands of primary care [33]. Future prospective studies of these and other systems changes, as well as national initiatives such as JCAHO-sponsored performance measures and financial practice incentives, may help elucidate the most effective means of delivering this valuable preventive service in primary care.

## Competing interests

No financial disclosures or other conflicts of interest were reported by the authors of this paper.

## Authors’ contributions

JAJ conducted the data analysis and led the writing of the manuscript. JPS designed the study and assisted with the writing of the manuscript. SS assisted with the study design and contributed to the development of the manuscript. MBH and RL both conducted data collection and provided feedback on the content of the manuscript. All authors read and approved the final manuscript.
